# Identification of serum biomarkers for colon cancer by proteomic analysis

**DOI:** 10.1038/sj.bjc.6603188

**Published:** 2006-06-06

**Authors:** D G Ward, N Suggett, Y Cheng, W Wei, H Johnson, L J Billingham, T Ismail, M J O Wakelam, P J Johnson, A Martin

**Affiliations:** 1 CR-UK Institute for Cancer Studies, University of Birmingham, Edgbaston, Birmingham B15 2TT, UK; 2 University Hospital Birmingham, Birmingham, UK

**Keywords:** colorectal cancer, SELDI, serum proteome, biomarker, proteomic, mass spectrometry

## Abstract

Colorectal cancer (CRC) is often diagnosed at a late stage with concomitant poor prognosis. Early detection greatly improves prognosis; however, the invasive, unpleasant and inconvenient nature of current diagnostic procedures limits their applicability. No serum-based test is currently of sufficient sensitivity or specificity for widespread use. In the best currently available blood test, carcinoembryonic antigen exhibits low sensitivity and specificity particularly in the setting of early disease. Hence, there is great need for new biomarkers for early detection of CRC. We have used surface-enhanced laser desorbtion/ionisation (SELDI) to investigate the serum proteome of 62 CRC patients and 31 noncancer subjects. We have identified proteins (complement C3a des-arg, *α*1-antitrypsin and transferrin) with diagnostic potential. Artificial neural networks trained using only the intensities of the SELDI peaks corresponding to identified proteins were able to classify the patients used in this study with 95% sensitivity and 91% specificity.

Colorectal cancer (CRC) is a major cause of worldwide morbidity and mortality and is the second most common cause of cancer death in Europe and the United States causing more than 50 000 deaths in the US and 16 000 in the UK each year (Greenlee *et al*, 2001; CRUK, 2004). CRC follows a gradual progression from benign polyps through early cancers to late and metastatic cancers (Jackman and Mayo, 1951; Tierney *et al*, 1990). Screening programmes for early diagnoses have resulted in a reduction in mortality (Newcomb *et al*, 1992; Selby *et al*, 1992; Muller and Sonnenberg, 1995) because survival decreases with increasing stage. Endoscopic examination of the colon remains the gold standard for diagnosis; however, this is invasive, unpleasant and carries associated risk of morbidity and mortality. Identification of high-risk patients using a less invasive test would decrease the number of such procedures required. Carcinoembryonic antigen (CEA) is of proven benefit in prognosis and follow-up, but has limited sensitivity (30–40%) for early CRC (Fletcher, 1986), whereas serial faecal occult blood testing is proven to reduce CRC mortality but suffers from significant false-negative and false-positive rates (Hardcastle *et al*, 1989; Mandel *et al*, 1993; Kronberg *et al*, 1996). Stool DNA analysis for multiple targets has shown sensitivity of 71–91% in preliminary studies and larger studies are underway (Ahlquist *et al*, 2000; Dong *et al*, 2001); however, a serum-based assay with equivalent sensitivity and specificity would be more acceptable to many patients.

Surface-enhanced laser desorption/ionisation (SELDI) mass spectrometry (MS) is a technology that can produce proteomic ‘fingerprints’ from biological samples using a relatively high-throughput platform. The sample is diluted in an appropriate buffer and applied to ‘proteinchip arrays’ coated with chromatographic surfaces (anion and cation exchange, reverse-phase and immobilised divalent metal ion surfaces). A proportion of the peptides/proteins in the sample bind to the chip surface and the rest of the proteins and any other nonbinding components are rinsed away. Following addition of an energy absorbing organic acid the proteins on the surface are ionised into the gas phase using a laser and analysed by time-of-flight mass spectrometry (i.e. according to their mass/charge ratio). Multivariate analysis can then be used to determine whether the intensities of the peaks in the SELDI spectra of different patient groups possess discriminatory ability. Studies have suggested the possible utility of SELDI analysis in diagnosing ovarian (Petricoin *et al*, 2002; Kozak *et al*, 2003), prostate (Adam *et al*, 2002; Qu *et al*, 2002; Banez *et al*, 2003), breast (Li *et al*, 2002), bladder (Vlahou *et al*, 2001), hepatic (Poon *et al*, 2003; Ward *et al*, 2006) and pancreatic cancer using serum. Concerns with several aspects of this approach have been raised, including potential bias in sample collection protocols, accuracy/resolution of the PBS IIc mass spectrometer employed in SELDI analysis, alignment of the detected peaks and over fitting of the data (Ransohoff, 2004). However, the potential benefits of a sensitive and reliable serum-based diagnostic test for a range of diseases, including cancer are so great that many efforts are being made to solve these problems.

More recently, SELDI and other MALDI-based approaches have been used to detect proteins that are differentially expressed between patient groups that can then be isolated and identified, often using MS/MS approaches (Le *et al*, 2005; Malik *et al*, 2005; Paradis *et al*, 2005). This information could be useful in the design of more specific diagnostic tests or inform us about the disease process.

In this report, we describe the analysis of noncancer and CRC samples by SELDI and identify proteins responsible for peaks which characterise the CRC samples and therefore have the potential to function as biomarkers.

## MATERIALS AND METHODS

### Patient/sample information

Serum samples were obtained from patients attending the University Hospital Birmingham rapid-access clinic for primary care referrals with suspected CRC, or from healthy volunteers. Ethical approval was obtained for sample collection and all patients gave informed consent. Blood was collected into standard hospital blood collection tubes and allowed to clot at 4°C for 1–2 h, then warmed to room temperature for 30 min before centrifugation (2500 **g** for 10 min) and the serum aliquoted and stored at −80°C. Cancers (62 samples) and controls (31 samples) were collected into identical tubes and processed in an identical manner.

The noncancer group consisted of 13 male and 18 female patients *vs* 36 male and 26 female patients for CRC, aged 62.9±10.3 years for the noncancer *vs* 67.3±12.9 years for CRC. The CRC group contained 27 patients with localised disease (Dukes' A/B) and 35 patients with disseminated disease (Dukes' C/D). The noncancer group (31 samples) contained patients from the same rapid-access clinic (27) as the CRC patients (12 diverticular disease, 15 no abnormality detected) or healthy volunteers (4). The noncancers were predominantly individuals referred to the rapid-access clinic because of indicative symptoms that were determined to not have CRC and the cancers were individuals proven to have CRC by attending the same clinic. This represents the ‘real-world’ comparison presented to colon practitioners when diagnosing colon cancer, rather than healthy controls that may not constitute as relevant a comparison for a diagnostic test.

### SELDI analysis

Sera were analysed on Cu^2+^-loaded IMAC30 proteinchip arrays. The samples (including duplicates) were randomised with respect to position in the bioprocessor. IMAC proteinchip arrays were prepared by incubation with 100 mM CuSO_4_ for 5 min (50 *μ*l per spot) followed by a water rinse and 3 × 10 min washes with 200 *μ*l binding buffer (500 mM NaCl, 100 mM NaH_2_PO_4_/NaOH, pH 7.0). All sera were diluted five-fold in 9 M urea, 50 mM Tris/HCl, pH 9.0, 2% (w v^−1^) CHAPS, followed by a 10-fold dilution in binding buffer before the addition of 100 *μ*l diluted sample per well. Binding was allowed to proceed for 1 h at room temperature with shaking at 900 r.p.m. The proteinchip arrays were then washed four times using 200 *μ*l of binding buffer (10 min with shaking) followed by a water rinse. The proteinchip arrays were allowed to dry and 1 *μ*l of a 50% saturated solution of sinapinic acid in 50% acetonitrile, 0.5% trifluoroacetic acid (matrix solution) applied to each spot. After air drying, another 1 *μ*l of matrix solution was added and the spots air-dried before analysis in a PBS IIc SELDI-TOF equipped with an autoloader (Ciphergen, Biosystems Inc., Fremont, CA, USA). Spectra were collected over 0–20 and 0–200 kDa ranges (600 laser shots) using laser intensity settings of 165/185 (low range/high range). Spectra were externally calibrated using neurotensin, cytochrome *c*, myoglobin, chymotrypsinogen and bovine serum albumin (Sigma-Aldrich, Poole, Dorset, UK) and the intensities normalised using the total ion current. Spectra with a total ion current of less than 20% of the average for the experiment were excluded from the analysis. Peaks were detected automatically using Ciphergen proteinchip software (valley depth and peak height both set at two times the noise) and those peaks present in >10% of the spectra clustered using the Biomarker Wizard tool (manufacturer's default settings). Peak intensities for duplicate spectra were combined and compared between the noncancer and CRC groups using two-sample *t*-test and the area under the receiver operator characteristic (ROC) curve. Peaks found to be statistically significantly different between the groups were used to develop artificial neural networks (ANNs).

### Sample classification

Artificial neural networks were used to classify serum samples into cancer and noncancer as described previously (Ward *et al*, 2006). The feedforward neural networks consisted of three layers: an input layer, a hidden layer and an output layer. The number of input nodes was determined by the number of significant peaks from which the models were trained. The hidden layer connected the input and output layers and the number of nodes in this layer controlled the complexity and performance of the neural networks. The output layer consisted of a single node whose output was used to classify sample status, representing cancer or noncancer. The model had full connection from the input nodes to the hidden nodes and from the hidden nodes to the output node. All of the connection weights were randomly initialised in the range (−1, +1). The networks were trained using the back propagation algorithm and tested using 10-fold cross validation.

### Biomarker purification

The initial step in the purification of the 6.44, 6.64, 8.94 and 50.7 kDa peaks (based on a method validated by our group previously; Poon *et al*, 2003) was to dilute the serum (100 *μ*l) three-fold in 9 M urea, 50 mM Tris/HCl, pH 9 and 2% CHAPS buffer, and apply it to Q Ceramic HyperD F anion exchange beads (Pall, New York, USA) in spin-cup filters (Pierce, Rockford, Illinois, USA). The proteins that did not bind were collected by centrifugation. The beads were then washed sequentially with buffers at pH 7, 5, 4, and 3, and finally with 50% acetonitrile+0.5% trifluoroacetic acid and the eluates collected. The 8.94 kDa peak did not bind to the beads and the only additional purification step was SDS–PAGE. The 6.64 and 6.44 kDa peaks bound to the beads and eluted predominantly at pH 5. This fraction was applied to a C-18 reverse-phase column (Vydac, 4.6 × 300 mm) equilibrated in solvent A (0.1% TFA in water) and proteins eluted using a linear gradient of 0% solvent B (0.08% TFA in acetonitrile) to 100% B over 40 min at a flow rate of 0.5 ml min^−1^. The fractions were analysed by SELDI and those containing the 6.64 and 6.44 kDa peaks concentrated by vacuum evaporation and then separated by SDS–PAGE. The 50.7 kDa peak eluted at pH 4 and was also separated using reverse phase HPLC and analysed by SELDI but this time using the second dimension of a Beckman Coulter PF 2D protein purification system (see below).

The 79.1 kDa protein was purified using a Beckman Coulter PF 2D-automated two-dimensional chromatography system. Pooled noncancer and pooled CRC sera were run in triplicate on the system. The sera were diluted in the pH 8.5 ‘start buffer’ and the protein concentration measured (Pierce BCA protein assay). Total protein (2.5 mg) for each sample was applied separately to the first dimension (chromatofocusing) at a flow rate of 0.2 ml min^−1^, a pH gradient formed using ‘elution buffer’ (pH 4.0) and fractions collected every 0.3 pH unit. Fractions from this first dimension were diluted and applied to Cu^2+^-loaded IMAC proteinchip arrays and the fractions containing the 79.1 kDa peak determined. The second dimension consists of a monolithic C-18 reverse phase column (equilibrated in solvent A) used to fractionate sequentially each of the first dimension fractions. Proteins were eluted using a linear gradient of solvents A to B over 30 min at a flow rate of 0.75 ml min^−1^ and fractions collected. Proteins eluted from the second dimension separation were quantified by measuring the absorbance at 214 nm. The second dimension fractions derived from the first dimension fractions that contained the 79.1 kDa peak were again screened by SELDI and the fractions containing the 79.1 kDa peak were separated by SDS–PAGE.

The individual proteins from the purification schemes given above were processed for liquid chromatography-tandem mass spectrometry (LC-MS/MS). Briefly, the band of interest was excised, washed, reduced using 50 mM dithiothreitol, alkylated with 100 mM iodoacetamide and digested overnight with 250 ng modified trypsin (Promega, Madison, Wisconsin, USA). The LC-MS/MS analysis was performed using an LC Packings Ultimate HPLC system linked to a ThermoFinnigan LCQ Deca XP Plus ion-trap mass spectrometer via a nanospray interface fitted with a metal emitter tip. The peptides were separated using a 180 *μ*M ID ThermoFinnigan BioBasic C-18 reverse phase-column run at 1.25 *μ*l min^−1^ that was equilibrated with 95% solvent C (5% acetonitrile in water/0.1% formic acid) and 5% solvent D (95% acetonitrile in water/0.1% formic acid) and eluted with a gradient of 5–37.5% D over 25 min. The ion-trap was set to detect positively charged ions using a spray voltage of 2.5 kV and an automated data-dependent MS/MS analysis performed on the five most abundant ion species from each MS full scan before another MS full scan was performed. Peptides were analysed a maximum of two times and were then placed on an exclusion list for 1 min. The MS/MS spectra were searched against an NCBI nonredundant human database using TurboSequest as part of the Bioworks 3.1 suite of programmes. All analyses were carried out at least twice and only proteins with multiple peptides detected, using XCorr cutoff values of 2.5 for triply, 2.0 for doubly and 1.5 for singly charged ions, are given.

Western blots were performed on samples after SDS–PAGE using Immobilon PVDF membrane. The antibodies used were, anti-C3a antibody from Research Diagnostics Inc., Concorde, Massachusetts (catalogue number RDI-PRO61018), anti-apolipoprotein C1 from Chemicon International Inc., Temecula California (catalogue number MAB 1064), anti-*α*1-antitrypsin from Abcam, Cambridgeshire, UK (catalogue number ab9399) and anti-transferrin from Abcam (ab1223). Where immunodepletions were performed the antibodies were pre-bound to Protein-G beads before addition to the serum. The depleted sera were retained following removal of the beads and associated proteins. The beads were washed and eluted with 50% acetonitrile/0.5% trifluoacetic acid. Control incubations using irrelevant antibodies or no antibody were performed to confirm specificity.

### Immunoassays

The C3a ELISA was carried out using a kit from Research Diagnostics Inc. according to the manufacturer's instructions. Carcinoembryonic antigen was measured using a Roche Modular Immunoassay E170 analyser using the manufacturer's reagents and recommended methodology at the Worcestershire Acute Hospitals Trust.

## RESULTS

A SELDI analysis of CRC patient's sera for the low range (0–20 000 *m*/*z*) and high range (0–200 000 *m*/*z*) were performed and two-sample *t*-tests were carried out to determine which peak intensities were significantly different in the noncancer *vs* CRC groups (see Table 1) using the SELDI spectra from individual patients. Varying numbers of the most significant peaks were then used to develop ANNs to discriminate between cancer and noncancer with 10-fold cross-validation. The ANNs developed using the seven most significant peaks performed best giving a sensitivity of 94% and specificity of 96%.

A pooled CRC sample (containing serum from 46 individuals) and a pooled noncancer sample (26 individuals) were analysed in quadruplicate on 10 IMAC proteinchip arrays prepared at intervals over an 11-week period. This experiment was designed to assess the reproducibility of the analysis and also to confirm proteomic features characteristic of colon cancer. The average intrachip coefficient of variation (CV) for the peak intensities in both samples was 18% and the average CV for both samples across all 10 proteinchip arrays was 25%. In addition, the Euclidean distance and correlation coefficient (always >0.97) between the peak heights on each of the 10 proteinchip arrays, relative to the first proteinchip array, did not show any trends across the experiment. These data demonstrate that SELDI spectra are reproducible over extended periods if materials and methods are not changed. The results in Table 2 show the peaks that are detected as significantly different between the two pooled samples (here the *P*-values do not reflect biological but simply experimental variation). Many of these peaks are the same (marked with ^*^) as those found to be significantly different in the analysis of the individual samples (Table 1). The significant proteomic features common to both experiments were considered as suitable candidates for purification and identification.

The initial strategy to isolate the peaks of interest was based on the serum fractionation protocol used by Poon *et al* (2003). The sera were diluted in a buffer designed to disrupt protein/protein interactions (9 M urea and 2% CHAPS, pH 9). Under these conditions, proteins with a *p*I below pH 9 bind to anion exchange resin and those with a *p*I above pH 9 do not. The bound proteins can then be eluted by washing the resin sequentially with buffers of decreasing pH. The nonbinding sample and the various eluates were analysed using Cu^2+^-loaded IMAC30 proteinchip arrays and the fractions containing the peaks of interest determined.

**Figure 1:**
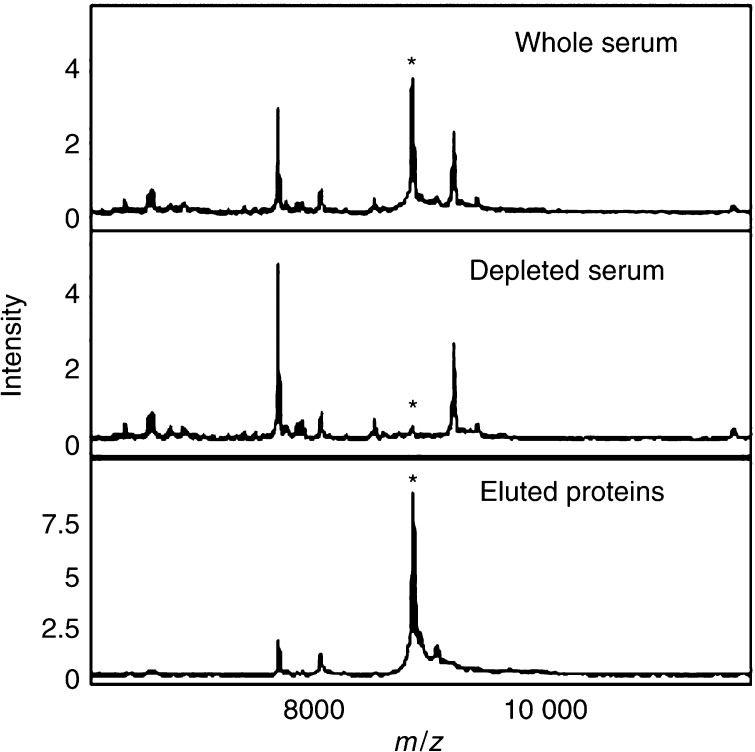
Immunodepletion of complement C3a des-arg. Serum was incubated with an anti-complement C3a des-arg mouse monoclonal antibody bound to protein G sepharose. The protein G sepharose was collected by centrifugation and the non-bound proteins (depleted serum) retained. The beads were washed and the bound proteins eluted. The starting serum (upper panel), non-bound proteins (middle panel) and eluted proteins (lower panel) were analysed using Cu^2+^-loaded IMAC proteinchip arrays.

The 8.94 kDa peak did not bind to the anion exchange resin at pH 9, and when the nonbound material was separated by SDS–PAGE, a differentially expressed band of approximately the expected mobility was seen (data not shown). In-gel digestion and LC-MS/MS analysis of this band detected four tryptic peptides (Table 3) derived from complement C3, a protein with a predicted mass of 185 kDa that is cleaved to produce complement C3a with a mass of 9095 Da. This protein has its C-terminal arginine residue removed to produce complement C3a des-arg with a mass of 8938 Da corresponding closely to the mass of the differentially detected peak in the SELDI analysis. All of the complement C3 tryptic peptides detected were from within the complement C3a des-arg sequence and represent 27% of the C3a des-arg. Complement C3a des-arg has a predicted *p*I of 9.3 in agreement with the observation that this molecule did not bind to the anion exchange resin at pH 9. The identity of the SELDI peak at 8.94 kDa was verified using an anti-complement C3a antibody to deplete complement C3a from serum. The SELDI peak at 8.94 kDa was specifically removed from the serum by the antibody treatment and was subsequently recovered following elution from the antibody (Figure 1) confirming the identity of the 8.94 kDa peak as complement C3a des-arg.

**Figure 2:**
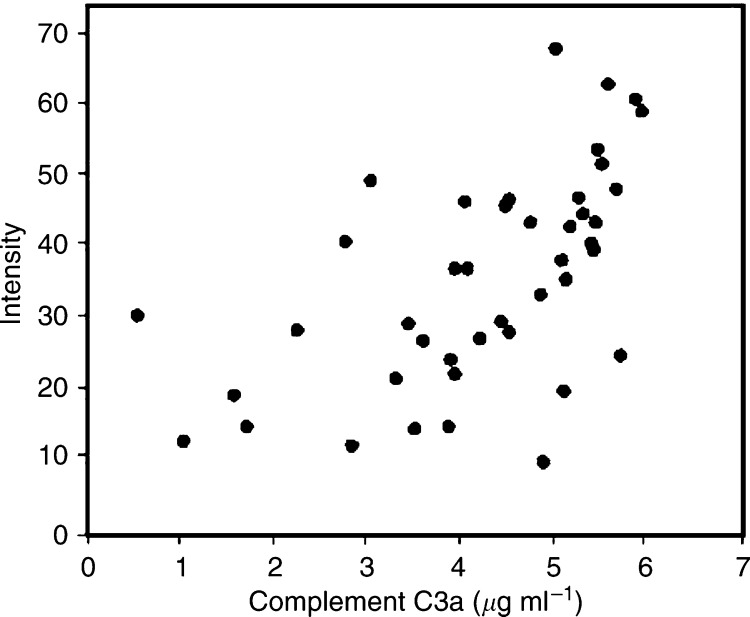
Comparison of the SELDI peak intensity at 8940 *m*/*z* and the complement C3a levels in serum. The complement C3a des-arg level was measured using an ELISA kit from Research Diagnostics Inc. using the manufacturer's instructions. The results shown are the concentration of C3a (*μ*g ml^−1^) plotted against SELDI peak intensity in the same sample.

In order to relate the SELDI peak intensities with complement C3a des-arg abundance, a complement C3a des-arg ELISA was performed. The result in Figure 2 shows a correlation between the SELDI peak intensity and the ELISA determined C3a abundance.

**Figure 3:**
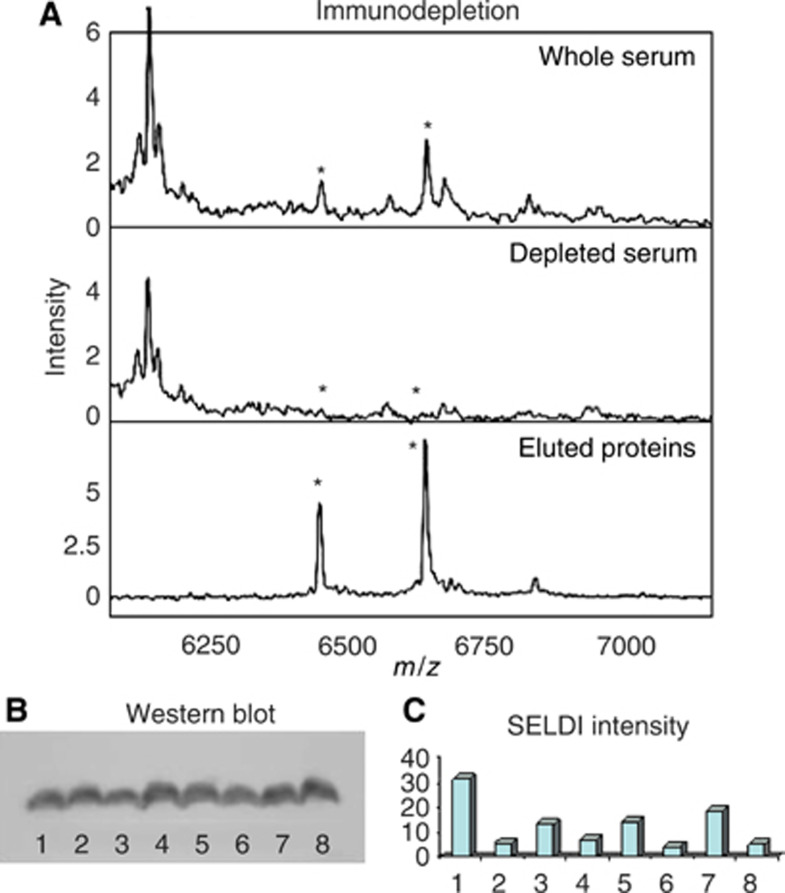
Immunodepletion of apolipoprotein C1 and a comparison of the intensity of the peak at 6640 *m*/*z* with Western blot analysis of apolipoprotein C1. (**A**) Serum was depleted using an anti-apolipoprotein C1 mouse monoclonal antibody using the same protocol given for the immunodepletion of complement C3a given in Figure 1. (**B**) A Western blot using an anti-apolipoprotein C1 antibody. The whole length and truncated forms of apolipoprotein C1 differ in mass by 198 Da and are not resolved by the SDS–PAGE so only a single band is observed. The samples were selected on the basis of a high or low SELDI peak height, as shown in (**C**). The SELDI peak intensity at 6640 *m*/*z* using Cu^2+^-loaded IMAC proteinchip arrays for the same samples for the Western blot is shown. The 6440 *m*/*z* peak displayed a similar pattern of intensities as the 6640 *m*/*z* peak (results not shown).

The 6.44 and 6.64 kDa peaks coeluted from the ceramic HyperD F anion exchange resin in the pH 7–4 fractions. The sample containing the most intense peaks (pH 5 elution) was separated using RP–HPLC and the eluted fractions screened by SELDI. The fractions containing the 6.44 and 6.64 kDa peaks were separated using SDS–PAGE and a band with the correct mobility excised, digested and analysed using LC-MS/MS. Six peptides covering 56% of the sequence of apolipoprotein C1 were detected (Table 3). Apolipoprotein C1 has a predicted sequence mass of 6631 Da and an additional truncated form with threonine and proline removed from the N-terminus with a mass of 6433 Da has been reported (Bondarenko *et al*, 1999). Immunodepletion of serum using an anti-apolipoprotein C1 antibody removed both the 6.44 and 6.64 kDa peaks, which were recovered following elution from the antibody (Figure 3A). Western blot analysis of serum samples (Figure 3B) did not detect any differences in apolipoprotein C1 concentration.

**Figure 4:**
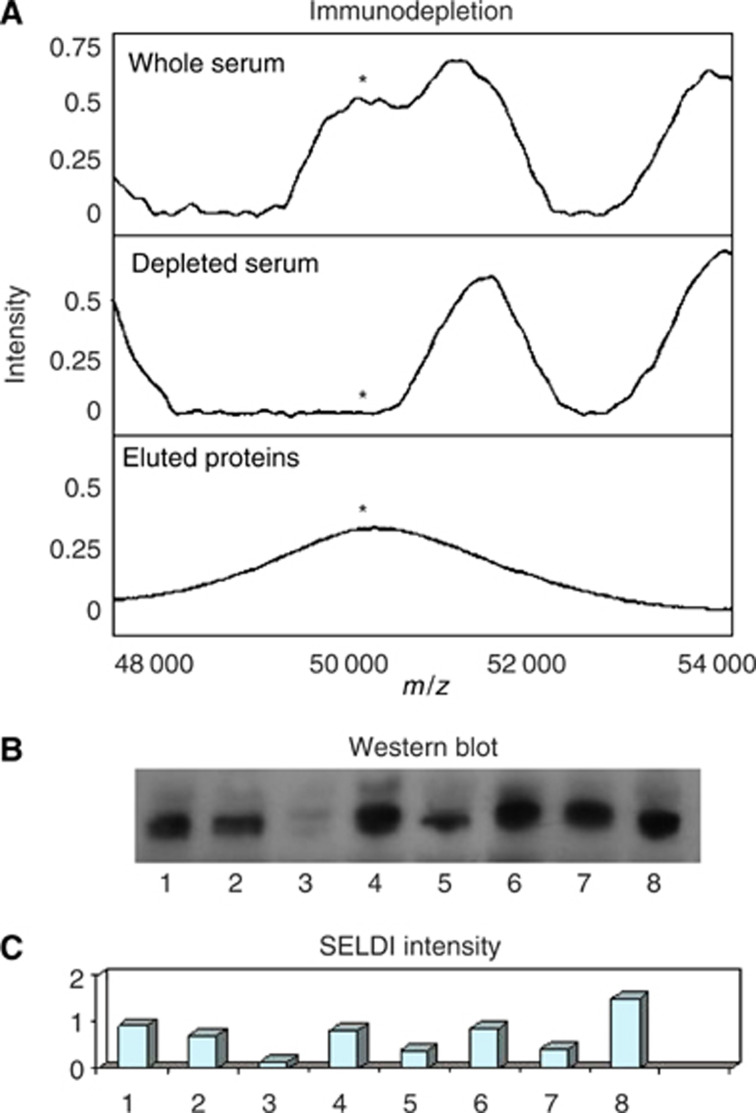
Immunodepletion of *α*1-antitrypsin and a comparison of the intensity of the peak at 50 700 *m*/*z* with Western blot analysis of *α*1-antitrypsin. (**A**) An immunodepletion of *α*1-antitrypsin using a mouse monoclonal antibody was performed using the same strategy given in Figure 1 for complement C3a. (**B**) Western blot analysis of eight samples (**C**) Corresponding SELDI intensity for the 50 700 *m*/*z* peak. The samples used were selected on the basis of the 50 700 *m*/*z* peak intensity to asses the correlation between Western blot analysis and SELDI peak hight.

The 50.7 kDa biomarker eluted from the ceramic HyperD F anion exchange resin at pH 4 and was further purified by RP-HPLC and the relevant fractions separated by SDS–PAGE. A band migrating with an apparent MW of 50.7 kDa was excised and trypsinised, and the peptides harvested. LC-MS/MS analysis detected 27 unique peptides from *α*1-antitrypsin (50% sequence coverage) and seven unique peptides from *α*1-antichymotrypsin (20% sequence coverage). Both of these proteins are glycosylated, have similar molecular masses and isoelectric points and their co-purification has been reported previously (Koomen *et al*, 2005). It seemed likely that *α*1-antitrypsin is the major contributor to the 50.7 kDa biomarker peak and this was confirmed by Western blot and immunodepletion. Furthermore, the SELDI peak intensity and Western blot staining (Figure 4) correlate, indicating that the concentration of *α*1-antitrypsin is higher in the serum of CRC patients in this study, as detected by SELDI peak height.

**Figure 5:**
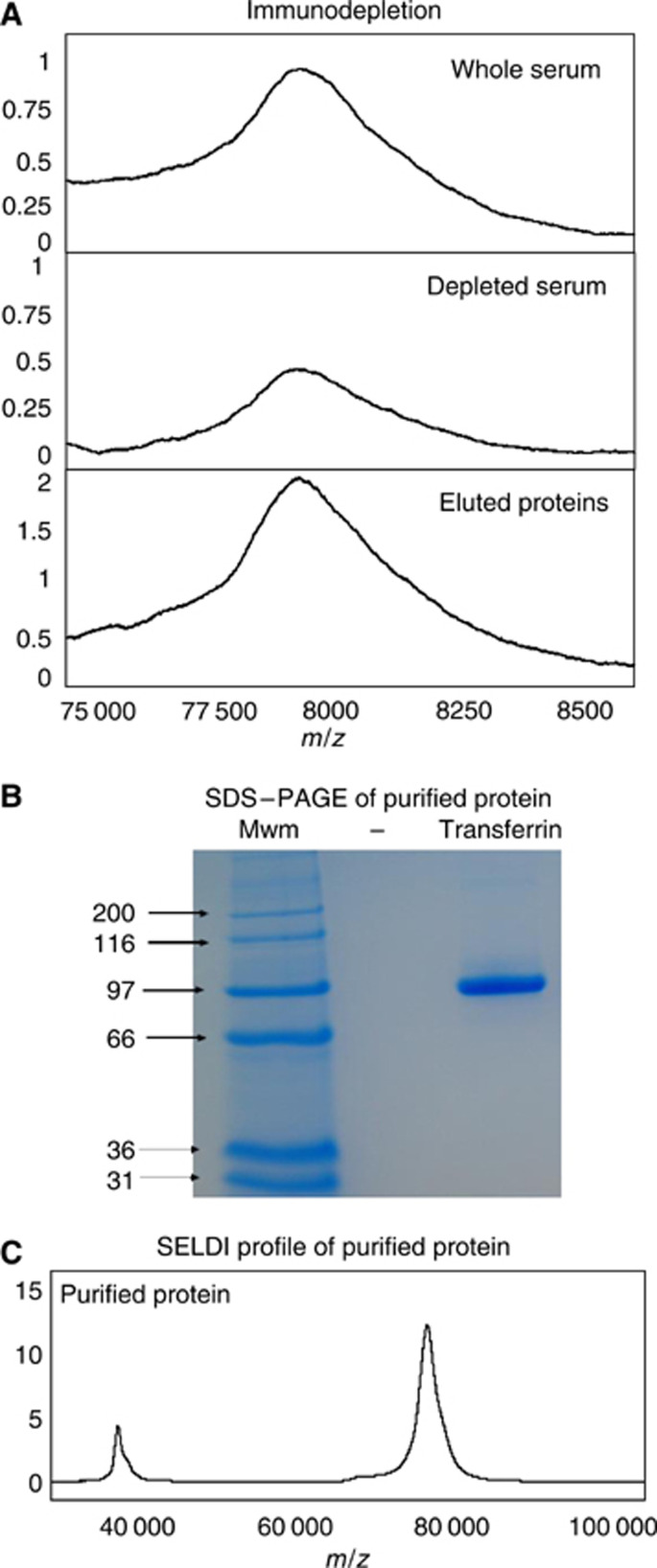
Immunodepletion and purification of transferrin. (**A**) An immunodepletion of transferrin employing an identical protocol to that in Figure 1. The peak of 79100 *m*/*z* was purified by automated 2D HPLC and the fractions monitored using SELDI. A Coomassie-stained SDS–PAGE gel shows a clear single band at approximately 80 kDa (**B**). (**C**) SELDI spectrum of this purified protein with a peak at the predicted size of 79 100 *m*/*z* in addition to an another at 39 900 *m*/*z*.

The 79.1 kDa biomarker was purified by 2D liquid chromatography (Beckman-Coulter PF 2D system) resulting in an essentially pure 79.1 kDa protein that binds to the IMAC proteinchip array (Figure 5B). This protein was identified as transferrin (44 unique peptides giving 57% sequence coverage). Absorbance at 214 nm of the transferrin revealed a modest increase in concentration in the pooled CRC sample relative to the pooled normal sample in agreement with the SELDI analysis, and Figure 5A shows the SELDI analysis of the immunodepletion/elution. The anti-transferrin antibody decreased the intensity of the 79.1 kDa peak, which was specifically detected in the eluted material.

The SELDI spectra obtained during the purification of the transferrin showed a co-purifying peak of 39 900 *m*/*z* that is not seen in the stained gel (Figure 5B and C), which corresponds to the *m*/*z* of a differentially expressed peak in the SELDI IMAC chip analysis of individual and pooled samples (Tables 1 and 2). When the transferrin immunodepletion was performed, the SELDI peak at 39.9 kDa was also decreased and recovered in the eluted material (not shown) and commercially available purified transferrin displayed two peaks of approximately 79.1 and 39.9 kDa (not shown). It is possible therefore that the 39.9 kDa peak is the doubly charged transferrin ion, but the mass of the ion does not correspond accurately to the predicted *m*/*z* value, which may be due to the relatively low mass accuracy of the PBS II analyser.

### Sample classification using only the six SELDI peaks corresponding to apolipoprotein C1, complement C3a des-arg, *α*1-antitrypsin and transferrin

Using unidentified peaks in SELDI spectra to classify patients is essentially a ‘black box’ approach. Having identified six of the peaks (the 6.44, 6.64, 8.94, 50.7, 79.1 and the potentially doubly charged version of the 79.1 kDa protein at 39.9 kDa), an ANN was developed using only these peaks. This ANN was able to classify the samples with 95% sensitivity and 91% specificity (10-fold cross validation).

### Comparison and combination of ANNs with CEA

Carcinoembryonic antigen was measured in all of the samples, and using the manufacture's recommended cutoff level of 4 ng ml^−1^, the sensitivity and specificity obtained was 53 and 93%, respectively. Furthermore, inclusion of the CEA data in an ANN with the SELDI data for the six SELDI peaks identified here did not improve the ANN for the SELDI peaks alone.

## DISCUSSION

The results presented in this paper demonstrate that SELDI analysis of CRC serum, compared to noncancer detects an altered intensity in a number of characteristic peaks which, when analysed by ANNs, have sensitivities and specificities in excess of 90%. This work identified some of these peaks as transferrin, *α*1-antitrypsin, complement C3a des-arg and apolipoprotein C1. A similar use of SELDI by Chen *et al* (2004), Guang *et al* (2004) and Yu *et al* (2004) suggested that SELDI profiling could be more sensitive than CEA analysis in diagnosing CRC. The data in this paper support this, but, additionally, identifies potential biomarkers, which require validation with large numbers of patients and if successful could point to the development of more widely applicable immunoassays.

In this study, we screened for biomarkers in two distinct ways: (1) multiple measurements of two pooled samples containing serum from many noncancer controls or CRC patients and (2) duplicate measurements of serum from many noncancer individuals or patients with CRC. The first approach gave a thorough investigation of how reproducible SELDI peak heights were during this study: we find the intrachip CV to be within the manufacturer's specification and an overall CV of 25%. Using the second approach, we collected duplicate SELDI spectra for each serum sample from 31 noncancer controls and 62 CRC patients. Having separate data on all the individuals in the study allowed us to assess diagnostic utility (area under the ROC curve, see Table 1) and to develop algorithms for patient classification. It can be seen that regardless of which of these two methods is employed, the majority of the peaks identified as significantly different in the CRC patients were the same (Tables 1 and 2).

The four proteins that we have identified as underlying six of the SELDI peaks with diagnostic potential are all classical serum proteins; however, this need not exclude their use as cancer biomarkers (Poon *et al*, 2001). The pair of peaks detected at 6440 and 6640 *m*/*z* were shown to be full-length apolipoprotein C1 and a truncated version that has been described previously (Bondarenko *et al*, 1999). Both of the peaks at 6440 and 6640 *m*/*z* were detected at decreased intensities in the CRC samples when the IMAC proteinchip array was employed (Tables 1 and 2). However, when the H50, Q10 and CM10 proteinchip arrays were used no differences in intensities were detected (results not shown). Furthermore, a Western blot for apolipoprotein C1 did not detect any difference between samples selected (Figure 3) on the basis of the intensity of the 6440 and 6640 *m*/*z* peaks determined using the IMAC proteinchip array. The reason for this is not clear but may be related to competition for binding at the retentate chromatography step and/or suppression of ionisation during the ionisation/desorption step. Presumably, the binding of these proteins to the IMAC proteinchip array and/or the ionisation/desorption step is influenced by underlying biochemical changes in one of the sample groups that do not interfere with the other proteinchip array types. This need not exclude the use of these discriminatory peaks in the development of ANNs to diagnose cancer if the observed differences are suitable for the purpose.

The identification of the peak at 8940 *m*/*z*, as complement C3a des-arg using MS/MS analysis was confirmed using an immunodepletion approach (Figure 1). Chen *et al* (2004) also detected an elevation of a peak at 8930 *m*/*z* in samples from colon cancer patients that may be the same protein. A peak of similar mass was identified as apolipoprotein A-II in prostate cancer samples (Malik *et al*, 2005) and as a fragment of vitronectin in hepatocellular carcinoma samples (Paradis *et al*, 2005), underlining the need to validate identifications using independent assays. The increased level of complement C3a des-arg seen in the CRC patient sera suggested an increased level of complement activation indicative of inflammation. Complement C3a is highly biologically active, binding to mast cells and basophils and triggering release of their vasoactive contents (the des-arg form represents a stable inactivated form of complement C3a). The elevated level of complement C3a des-arg in the serum of CRC patients may reflect an immune response to the tumour, or possibly *in vitro* complement activation (Mollnes *et al*, 1988). This is unlikely to be a problem in this study as all samples were handled in an identical manner and therefore any differences in *in vitro* complement activation should reflect the state of the complement system in the samples. The complement C3a ELISA assay shows that the SELDI intensity reflects the serum concentration but the two measurements were not absolutely comparable. The antibody used in the ELISA recognises the C-terminus of the peptide and hence will react with any complement C3 cleaved at this site, whereas the SELDI peak will only report on complement C3a des-arg (if, e.g., N-terminally truncated forms existed, then these would not contribute to the peak at 8940 *m*/*z*).

The levels of *α*1-antitrypsin determined by SELDI and Western blot (Figure 4) correlate well, indicating that *α*1-antitrypsin is elevated in the CRC patients in this study. Koomen *et al* (2005) recently reported that a broad SELDI peak around 51.5 kDa was differentially detected in serum from pancreatic cancer patients compared to controls. This peak was found to contain *α*1-antitrypsin, *α*1-antichymotrypsin (as observed here) and haptoglobin. Measurement of the haptoglobin levels did not show a difference between the control and cancer patients, but as multiple proteins were found in the peak it is quite possible that the difference was owing to an altered level of one or both of the other proteins, as we show here for *α*1-antitrypsin. Like the elevation in complement C3a des-arg, this suggests that an inflammatory response to the tumour is occurring. As such, it is unlikely that either protein would show high specificity for CRC (Koomen *et al*, 2005); however, they may be candidates for multiplexed immunoassays combining sensitive and specific biomarkers.

Acute phase proteins are usually defined as proteins that change concentration by 25% or more in response to a range of inflammatory disorders. The majority of proteins increase in concentration but transferrin is one that decreases. Here, we detect an increase in a peak of approximately 79.1 kDa in the serum of CRC patients compared to controls that was identified as transferrin (Figure 5). The primary function of transferrin is to transport iron around the body. Elevated body iron stores have been proposed to correlate with an increased risk of colon cancer and an increased proportion of transferrin loaded with iron has been linked with an increased cancer incidence, particularly in individuals who have a high intake of iron (Weinberg, 1994; Nelson, 2001; Mainous *et al*, 2005). It is not clear why the transferrin concentration is increased in the serum of colon cancer patients as the predicted response to inflammation is a decrease for this protein. Clearly, the cancer process is more complex than inflammation alone, and as iron appears to play a role(s) in cancer biology, it is possible that the increase in transferrin concentration observed in the serum of CRC patients is not an acute phase response.

In conclusion, proteomic profiling of serum from CRC patients and noncancer individuals, combined with the use of ANNs, can diagnose CRC with 94% sensitivity and 96% specificity in our cohort of patients. We have identified four proteins underlying six of the SELDI peaks that are significantly different between the noncancer controls and CRC patients. The proteins identified are common serum proteins and changes in their concentrations most likely reflect epiphenomena rather than secretion by cancer cells. Nonetheless, ANNs trained with just the SELDI peaks from these proteins are remarkably good at discriminating CRC, outperforming CEA.

## Figures and Tables

**Table 1:** Significant proteomic features from individual serum samples

**Peak (*m/z*)**	***P* (*t*-test)**	**AUC**	**Fold change**
4790	6.0 × 10^−6^	0.786	0.67
50 700	7.1 × 10^−6^	0.798	1.71
8940	0.00020	0.739	1.48
6440	0.00026	0.705	0.68
6640	0.00057	0.690	0.72
123 000	0.00065	0.712	0.75
4290	0.00077	0.701	0.67
8150	0.0014	0.682	1.31
76 000	0.0024	0.678	1.37
8760	0.0035	0.721	0.62
4480	0.0039	0.685	1.55
79 100	0.0043	0.676	1.21
39 900	0.0052	0.738	1.38

AUC, area under the ROC curve.

SELDI peaks significantly different in the sera of CRC patients. Serum samples from control and cancer patients were analysed in duplicate using Cu^2+^-loaded IMAC proteinchip arrays. The peak intensities between controls and cancer were compared and the fold change (cancer relative to controls) and significance are given. ROC curves for the significant peaks (*P*>0.05) were constructed and the area under the curve for each peak is shown.

**Table 2:** Significant peaks from the analysis of pooled samples

**Peak (*m/z*)**	***P* (*t*-test)**	**Fold change**
8150^*^	1.4 × 10^−12^	1.21
39 900^*^	8.9 × 10^−12^	1.56
79 100^*^	1.0 × 10^−9^	1.32
50 700^*^	3.9 × 10^−9^	1.31
11 530	1.0 × 10^−7^	3.01
9000	4.3 × 10^−7^	1.22
11 690	1.3 × 10^−6^	2.20
2285	6.9 × 10^−6^	0.72
4290^*^	1.8 × 10^−5^	0.75
5920	2.3 × 10^−5^	0.87
8940^*^	0.00028	1.20
7940	0.00039	1.09
4480^*^	0.00050	1.19
6640^*^	0.00076	0.79
3970	0.0012	1.20
6440^*^	0.0016	0.78

Pooled control and cancer samples were analysed 40 times using Cu^2+^-loaded IMAC proteinchip array. The peak intensities for the samples were compared and the significantly different peaks (*P*>0.05) are listed along with the *P*-value and fold change. Peaks marked with ^*^ are those that are also significantly different in the SELDI profiles of the individual samples given in Table 1.

**Table 3:** Tryptic peptides used to identify the 6.44/6.64 and 8.94 kDa biomarkers

**Peptide**	**MH+**	**ID**
FISLGEACK	1025.2	Complement C3a residues 42–50
FISLGEACKK	1153.4	Complement C3a residues 42–51
VFLDCCNYITELR	1703.9	Complement C3a residues 52–64
KVFLDCCNYITELR	1832.1	Complement C3a residues 51–64

TPDVSSALDK	1033.1	Apolipoprotein C1 residues 1–10
EFGNTLEDK	1053.1	Apolipoprotein C1 residues 13–21
EWFSETFQK	1202.3	Apolipoprotein C1 residues 40–48
TPDVSSALDKLK	1274.5	Apolipoprotein C1 residues 1–12
LKEFGNTLEDK	1294.4	Apolipoprotein C1 residues 11–21
MREWFESTFQK	1489.7	Apolipoprotein C1 residues 38–48

Partially purified proteins were separated using SDS-PAGE and the relevant gel slice excised, reduced, alkylated and trypsinised. The peptides were collected and subjected to LC-MS/MS analysis followed by a database search to identify the peptides. The upper panel shows the peptides derived from complement C3a and the lower panel the peptides from apolipoprotein C1.
